# Impact of an antimicrobial stewardship program on colistin resistance in a tertiary care hospital in Pakistan: a quasi-experimental study

**DOI:** 10.3205/id000097

**Published:** 2025-11-20

**Authors:** Memoona Irshad, Sher Muhammad Sethi, Bushra Jamil, Seema Irfan, Safia Awan, Shamim Raza, Aafia Zafar

**Affiliations:** 1 Medicine Department, Pakistan Kidney and Liver Institute Hospital, Lahore Lahore; 2 Medicine Department, Aga Khan University Hospital, Karachi, Pakistan; 3 Microbiology Department, Aga Khan University Hospital, Karachi, Pakistan; 4 Antimicrobial Stewardship Program, Aga Khan University Hospital, Karachi, Pakistan

**Keywords:** antimicrobial stewardship, colistin resistance, gram-negative infections, Klebsiella pneumoniae, multidrug-resistant organisms

## Abstract

**Background:**

Colistin is a last-resort antibiotic used against infections caused by multidrug-resistant gram-negative organisms, particularly carbapenem-resistant strains. Rising resistance to colistin is a significant global concern. To address this, an Antimicrobial Stewardship (AMS) Program was introduced in our hospital, including pre-authorization protocols for colistin use.

**Objective:**

To evaluate the prevalence of colistin-resistant organisms and determine the impact of AMS implementation on their occurrence and associated clinical outcomes.

**Methods:**

We conducted a quasi-experimental study at a tertiary care hospital in Pakistan, comparing data from 18 months before and after AMS implementation. Adult patients (>18 years) with confirmed infections due to colistin-resistant *Klebsiella pneumoniae*, Pseudomonas aeruginosa, or Acinetobacter spp. were included. Clinical and microbiological data were analyzed to assess differences in organism prevalence, mortality, and hospital stay duration.

**Results:**

A total of 121 patients met inclusion criteria, with 45 (37.2%) in the pre-AMS period and 76 (62.8%) in the post-AMS period. *Klebsiella pneumoniae* was the most frequently isolated organism in both groups. The overall in-hospital mortality rate was 34%, and the average length of stay was approximately 20 days, with no significant differences between periods. Despite AMS implementation, colistin resistance prevalence did not decline.

**Conclusion:**

While the AMS facilitated better identification and documentation of colistin-resistant infections, it did not significantly reduce their prevalence or associated mortality. Strengthened stewardship measures, continuous compliance monitoring, and alternative therapeutic strategies are needed to curb rising colistin resistance in high-burden settings.

## Introduction

Antimicrobial resistance (AMR) has emerged as one of the most pressing threats to global health, with multidrug-resistant (MDR) gram-negative bacteria posing a particularly formidable challenge [1]. The overuse and misuse of antibiotics in both community and healthcare settings have accelerated resistance development, leaving clinicians with limited therapeutic options for serious infections [2]. Among the few remaining agents effective against carbapenem-resistant organisms is colistin, a polymyxin antibiotic that has been revived as a last-line treatment [3]. However, the rising incidence of colistin resistance in various pathogens raises serious concerns about the future of infection management in critically ill patients [4].

Colistin resistance is especially concerning due to its association with increased mortality, prolonged hospital stays, and the risk of nosocomial outbreaks [5]. Surveillance studies have shown an alarming trend in resistance to colistin globally, particularly in regions with high antimicrobial use and weak regulatory oversight [6], [7]. In this context, Antimicrobial Stewardship (AMS) Programs have gained recognition as essential tools to optimize antibiotic prescribing, limit inappropriate use, and slow the spread of resistance [8].

While there is growing global evidence of the effectiveness of AMS in reducing antibiotic consumption and resistance patterns, data specifically evaluating their impact on colistin resistance remain sparse [9]. This is particularly true in low- and middle-income countries (LMICs), including Pakistan, where antibiotic misuse is widespread, diagnostic capabilities may be limited, and AMS infrastructure is still evolving [8], [10]. Pakistan bears a heavy burden of MDR gram-negative infections, yet there is limited data assessing the prevalence of colistin-resistant organisms or the effectiveness of stewardship efforts in containing them [11].

Given the reliance on colistin as a critical last-resort therapy, understanding how stewardship measures influence resistance patterns is essential to inform clinical practice and policy. Moreover, data from local healthcare systems are needed to tailor AMS strategies that reflect real-world resource limitations and microbial epidemiology.

This study was therefore undertaken to evaluate the prevalence of colistin-resistant gram-negative organisms before and after the implementation of an Antimicrobial Stewardship Program in a tertiary care hospital in Pakistan. Additionally, we aimed to assess the clinical outcomes associated with these infections, including mortality and hospital stay duration, to determine whether AMS introduction had a measurable impact on infection burden and patient prognosis.

## Methods

### Study design and setting

This was a quasi-experimental, retrospective study conducted at the Aga Khan University Hospital (AKUH), a tertiary care teaching hospital located in Karachi, Pakistan. The study aimed to assess the prevalence of colistin-resistant gram-negative organisms before and after the implementation of an Antimicrobial Stewardship (AMS) Program. The AMS was formally introduced at AKUH in October 2017 and remains an ongoing, multidisciplinary institutional initiative.

### Study period and population

Data were collected over a total duration of 36 months, comprising 18 months before (April 1, 2016 to September 30, 2017) and 18 months after (October 1, 2017 to April 30, 2019) the implementation of the AMS. Adult patients aged ≥18 years with confirmed infections caused by colistin-resistant *Klebsiella* *pneumoniae*, *Pseudomonas* *aeruginosa*, or *Acinetobacter* spp. were included. Patients with colonization (without clinical signs of infection) were excluded based on a thorough review of clinical documentation, laboratory parameters, and radiological data.

### Description of the Antimicrobial Stewardship (AMS) Program

The AMS at AKUH is a structured, multidisciplinary program comprising infectious diseases (ID) specialists, clinical pharmacists, microbiologists, and infection control professionals. While the program oversees the rational use of several high-priority antibiotics including carbapenems, linezolid, vancomycin, and colistin but it has a specific focus on colistin due to its critical role in treating MDR gram-negative infections.

AMS interventions for colistin include:

- Mandatory pre-authorization: Prescribing colistin requires approval from an ID consultant prior to initiation, whether empirically or for culture-directed therapy.
- Prospective audit and feedback: Cases where colistin is initiated are reviewed regularly by the stewardship team to assess indication, dosing, duration, and potential de-escalation based on clinical response and microbiological data.
- Monitoring and reporting: The AMS routinely monitors antibiotic utilization, resistance trends, and guideline adherence, with regular feedback provided to clinical departments.

These interventions aim to optimize colistin use, limit unnecessary exposure, and reduce selection pressure contributing to resistance.

### Microbiological testing and identification

All isolates of *K. pneumoniae*, *P. aeruginosa*, and *Acinetobacter* spp. exhibiting resistance to colistin during the study period were included. Identification of isolates was performed using the Analytical Profile Index (API 20E/NE, bioMérieux, France). For colistin susceptibility testing, the VITEK^®^ 2 system (bioMérieux, France) was used until June 2018. Subsequently, the Broth Microdilution (BMD) method, as recommended by the Clinical and Laboratory Standards Institute (CLSI), was adopted.

In our laboratory, BMD is the standard method used for determining colistin minimum inhibition concentration (MIC). The procedure is performed using microtiter plates with cation-adjusted Mueller–Hinton broth, following standardized protocols. Results are read after 18–24 hours of incubation, similar to the timeframe used for disc diffusion. Growth is assessed based on visible button formation or haziness in the wells, with absence of haziness indicating no growth. The MIC is interpreted as one dilution higher than the last well showing visible growth. For example, if growth is observed up to the well containing 1 µg/mL, the reported MIC is recorded as 2 µg/mL.

### Data collection and variables

Data were extracted from the HIMS database and microbiology laboratory records. Variables collected included patient demographics, site and source of infection, comorbid conditions (assessed via Charlson Comorbidity Index), severity of illness (measured by qSOFA score), length of hospital stay, need for surgical intervention, and patient outcomes (discharge, death, or discharge against medical advice).

### Outcomes

The primary outcome was the frequency of colistin-resistant gram-negative organisms before and after AMS implementation.

Secondary outcomes includes site of infection, in-hospital mortality, length of hospital stay and microbiological profile by organism type.

### Statistical analysis

Data were analyzed using SPSS version 23. Continuous variables were summarized as means and standard deviations (SD), while categorical variables were presented as frequencies and percentages. Comparisons between pre- and post-AMS groups were conducted using the Student’s t-test for continuous variables and the Chi-square test for categorical variables. A p-value of <0.05 was considered statistically significant.

### Ethical considerations

The study was reviewed and approved by the Ethics Review Committee of Aga Khan University Hospital (ERC No: 2019-1806-4547). Informed consent was waived due to the retrospective nature of the data collection.

## Results

A total of 270 patients were identified with colistin-resistant organisms during the 36-month study period (18 months before and after AMS implementation). After excluding 104 colonizers and 45 patients under 18 years of age, 121 adult patients with confirmed infections were included in the final analysis. In the pre-AMS period, the mean age was 53.7 years, while in the post-AMS period, it was 54.2 years (p-value: 0.87). The majority of patients were male (n: 90), with 31 in pre-AMS and 59 in post-AMS periods (p-value: 0.28). Pre- and post-AMS demographics in relation to age, gender, and length of stay are shown in Table 1 (Tab. 1).

**Figure 1:**
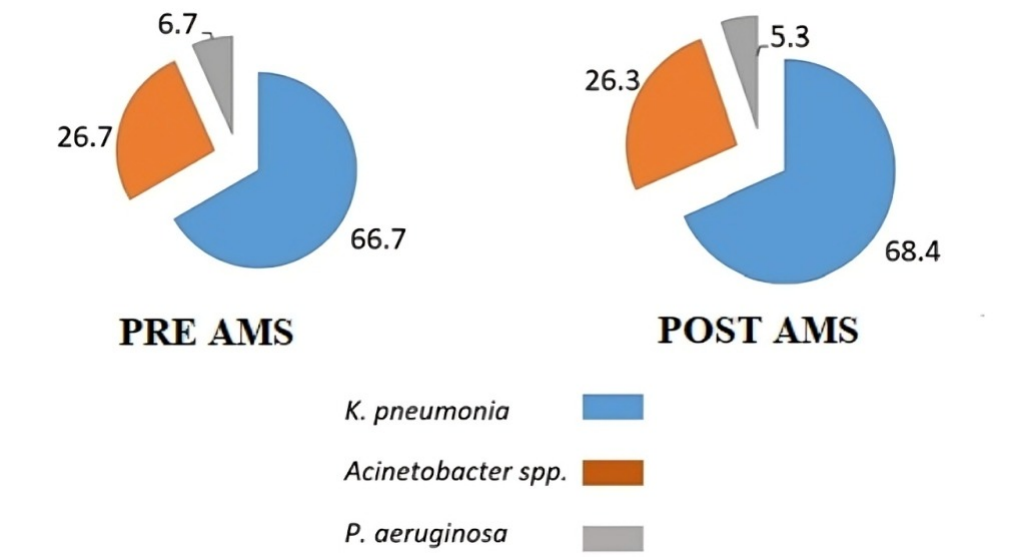
Distribution of colistin-resistant organisms in pre- and post-AMS period

Among the 121 patients, the most frequently isolated organism in both periods was *Klebsiella pneumoniae* (pre-AMS: 30; post-AMS: 52), followed by *Acinetobacter* spp. (pre-AMS: 12; post-AMS: 20) (Figure 1 (Fig. 1)). The most common infection site was pneumonia (pre: 14.0% and post: 32.2%). Other sites of infection include; tracheitis (pre: 6.7%, post: 6.6%), cystitis (pre: 11.1%, post: 9.2%), pyelonephritis (pre: 8.9%, post: 0%), skin and soft tissue infections (pre: 8.9%, post: 6.6%), intra-abdominal abscess (pre: 4.4%, post: 6.6%), surgical site infections (pre: 8.9%, post: 2.6%), central line associated infections (pre: 6.7%, post: 7.9%), cholangitis (pre: 0%, post: 3.9%), osteomyelitis (pre: 0%, post: 1.3%), necrotizing fasciitis (pre: 0%, post: 1.3%), nosocomial meningitis (pre: 0%, post: 1.3%), and neutropenia (pre: 2.2%, post: 0%). 

The overall in-hospital mortality rate was 34% (n=42), with *Klebsiella* *pneumoniae* infections contributing to the highest number of deaths. Pneumonia was also associated with higher mortality in 20 patients (16.5%). Table 2 (Tab. 2) summarizes outcomes stratified by organism type.

**Figure 2:**
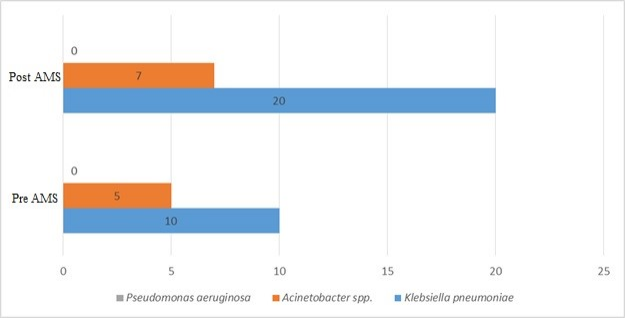
Comparison of mortality rates in pre- and post-AMS periods

In-hospital mortality remained high between the two time periods, increasing slightly from 33.3% in the pre-AMS period to 35.5% in the post-AMS period. This difference was not statistically significant and may reflect higher comorbidity and illness severity in the post-AMS group (Figure 2 (Fig. 2)).

**Figure 3:**
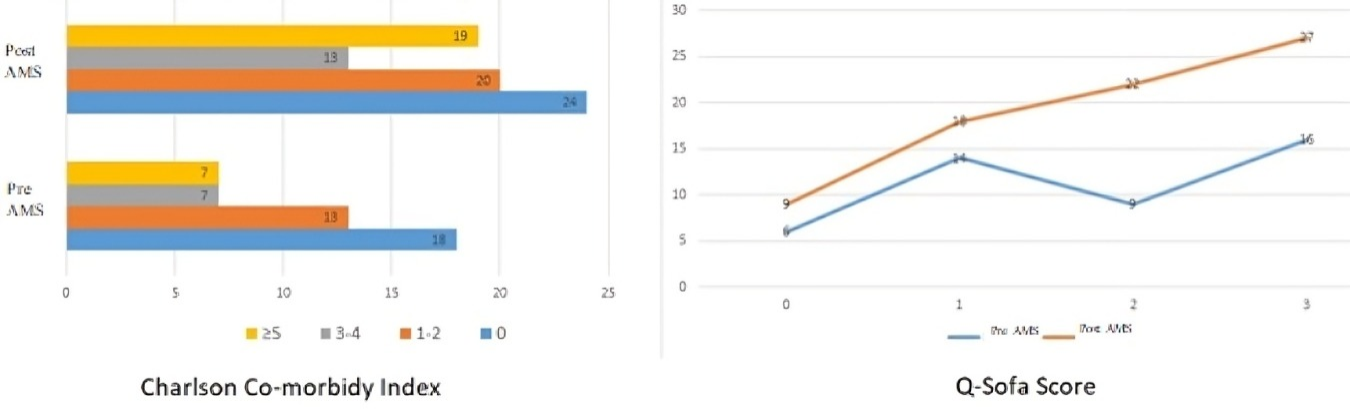
Comparison of average CCI and qSOFA scores between pre- and post-AMS groups

The average length of hospital stay was similar across both groups with 20.4±13.8 days in pre-AMS and 21.5±18.2 days in post-AMS periods. There was no statistically significant difference (p=0.74). Patients in the post-ASP group had higher Charlson Comorbidity Index (CCI) and qSOFA scores, suggesting a more critically ill population. This may explain the unchanged mortality despite AMS implementation (Figure 3 (Fig. 3)).

## Discussion

This study highlights a persistently high prevalence of colistin-resistant organisms in a tertiary care setting, despite the implementation of an AMS. Among the 121 patients analyzed, *Klebsiella* *pneumoniae* remained the predominant pathogen in both the pre- and post-AMS periods. Mortality rates were substantial and remained unchanged post-intervention. Similarly, average hospital stays were prolonged, indicating a sustained burden of resistant infections.

The AMS introduced at our institution focused on multiple high-priority antimicrobials, with specific measures targeting colistin use. While the program succeeded in standardizing prescribing practices through mandatory ID consultation, real-time audit, and controlled dispensing, its short-term impact on reducing colistin resistance was limited. This finding suggests that while stewardship structures are necessary, their effect may depend heavily on broader contextual factors including baseline resistance rates, patient acuity, and systemic adherence.

Multidrug-resistant organisms (MDROs), particularly *Klebsiella* *pneumoniae* and *Acinetobacter* spp., were predominant in our patient population [12]. This aligns with regional trends from South Asia, where overuse of broad-spectrum antibiotics and poor infection control infrastructure contribute to increasing resistance [13]. Similarly, in our study drug resistant *Klebsiella* was the predominant organism present in 82 out of 121 patients (67.7%) followed by *Acinetobacter* species present in 32 patients (26.4%). 

In terms of outcomes, both mortality and length of hospital stay were high in patients with colistin-resistant infections, irrespective of AMS status. The average hospital stay exceeded 20 days in both groups, and mortality remained around 34%. Post-AMS patients exhibited higher severity scores (CCI and qSOFA), suggesting that unchanged mortality rates may reflect a more critically ill population rather than AMS ineffectiveness. A study by Balkan et al. reported a 28-day mortality rate of 66%, with colistin resistance significantly associated with increased 28-day mortality and prolonged hospital stay [14].

A key strength of this study is its focused design and the inclusion of well-defined AMS metrics targeting colistin, a last-line agent. This adds value to the limited body of literature from LMICs evaluating stewardship effectiveness in the face of rising colistin resistance. The study also provides granular microbiological and clinical outcome data from a high-burden region.

However, several limitations must be acknowledged. First, the quasi-experimental design cannot fully account for confounders such as changes in infection control practices or external outbreaks. Second, while the study period post-AMS implementation was included, it may still have been insufficient to capture a significant shift in resistance patterns. Third, the sample size may not have been large enough to overcome the effect of potential confounders. Moreover, without parallel strengthening of infection prevention and control measures, AMS interventions alone may be limited in their impact, as clonal spread of resistant organisms could overshadow potential benefits of reducing selective pressure through restricted antibiotic use. Finally, as this was a single-center study, the findings may not be generalizable to other settings.

Future efforts should emphasize longer follow-up periods and multi-institutional collaboration to monitor AMS effectiveness across varying settings. There is also a need to integrate rapid diagnostic tools, resistance surveillance systems, and stricter infection prevention protocols. Policymakers must prioritize support for sustainable AMS infrastructure in resource-limited environments to counter the growing threat of antimicrobial resistance.

## Conclusion

Colistin resistance among gram-negative pathogens remains a significant challenge in hospital settings, with substantial mortality and prolonged hospital stays. Although AMS implementation facilitated more regulated antibiotic use, its immediate impact on resistance trends was limited. Continued investment in stewardship programs, coupled with broader infection control and surveillance initiatives, is essential to combat rising antimicrobial resistance in high-burden settings.

## Notes

### Competing interests

The authors declare that they have no competing interests.

## Figures and Tables

**Table 1:**
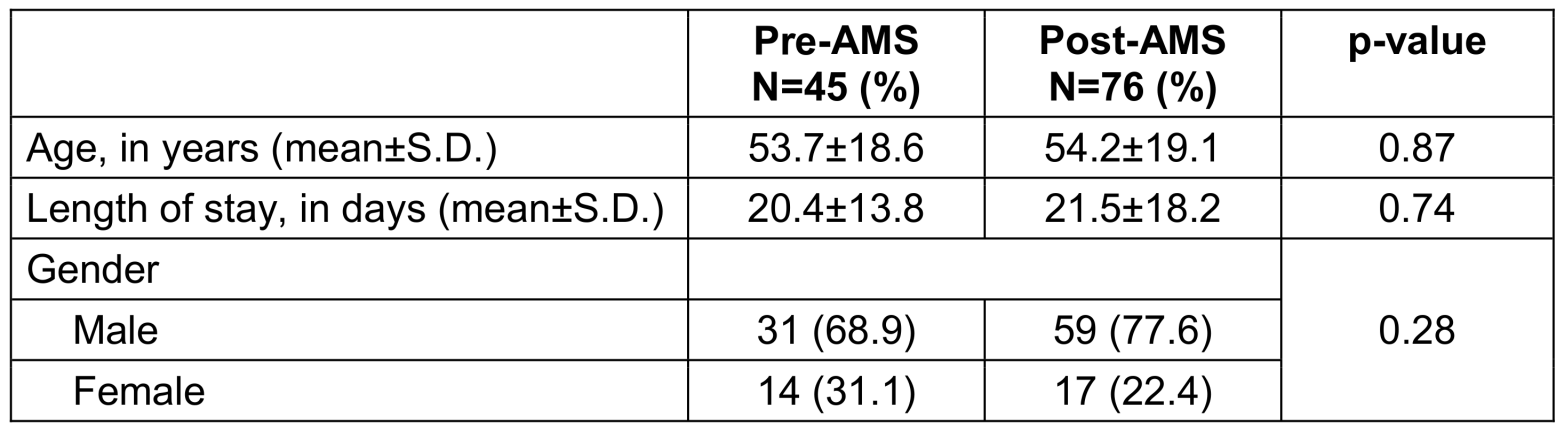
Demographics of patients admitted in pre- and post-AMS period

**Table 2:**
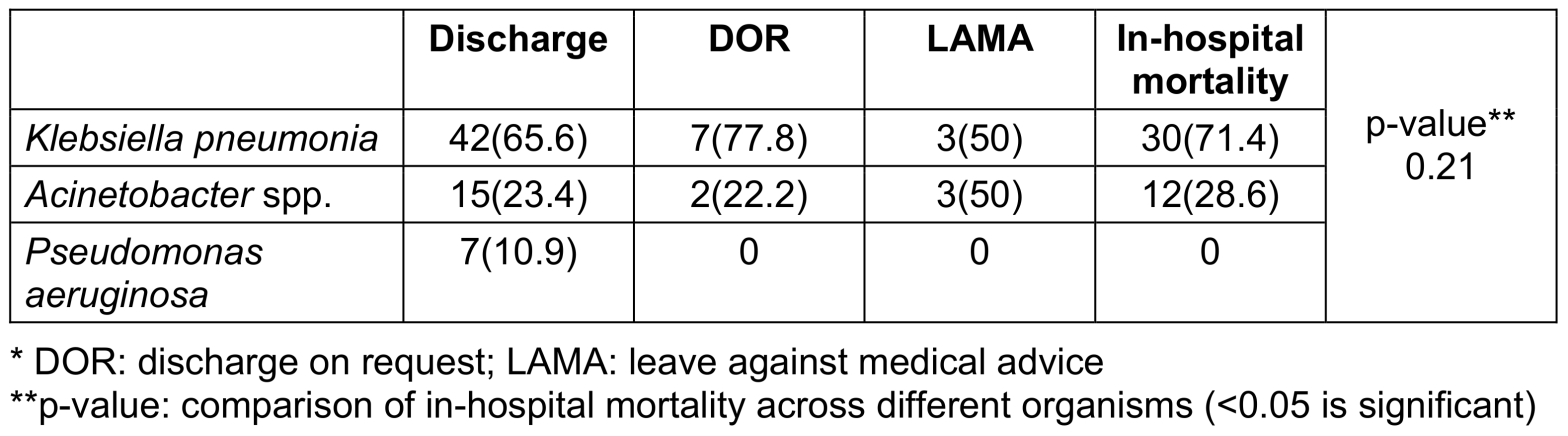
Outcomes by organism type

## References

[1] Salam MA, Al-Amin MY, Salam MT, Pawar JS, Akhter N, Rabaan AA, Alqumber MAA (2023). Antimicrobial Resistance: A Growing Serious Threat for Global Public Health. Healthcare (Basel).

[2] Muteeb G, Rehman MT, Shahwan M, Aatif M (2023). Origin of Antibiotics and Antibiotic Resistance, and Their Impacts on Drug Development: A Narrative Review. Pharmaceuticals (Basel).

[3] El-Sayed Ahmed MAE, Zhong LL, Shen C, Yang Y, Doi Y, Tian GB (2020). Colistin and its role in the Era of antibiotic resistance: an extended review (2000-2019). Emerg Microbes Infect.

[4] Sharma J, Sharma D, Singh A, Sunita K (2022). Colistin Resistance and Management of Drug Resistant Infections. Can J Infect Dis Med Microbiol.

[5] Llor C, Bjerrum L (2014). Antimicrobial resistance: risk associated with antibiotic overuse and initiatives to reduce the problem. Ther Adv Drug Saf.

[6] Yacouba A, Olowo-Okere A (2020). Global trends and current status in colistin resistance research: a bibliometric analysis (1973-2019). F1000Res.

[7] Mondal AH, Khare K, Saxena P, Debnath P, Mukhopadhyay K, Yadav D (2024). A Review on Colistin Resistance: An Antibiotic of Last Resort. Microorganisms.

[8] Atif M, Ihsan B, Malik I, Ahmad N, Saleem Z, Sehar A, Babar ZU (2021). Antibiotic stewardship program in Pakistan: a multicenter qualitative study exploring medical doctors' knowledge, perception and practices. BMC Infect Dis.

[9] Hassan J, El-Gemayel L, Bashour I, Kassem II, Hashmi MZ (2020). Chapter 10 - On the edge of a precipice: the global emergence and dissemination of plasmid-borne mcr genes that confer resistance to colistin, a last-resort antibiotic.

[10] Iskandar K, Molinier L, Hallit S, Sartelli M, Hardcastle TC, Haque M, Lugova H, Dhingra S, Sharma P, Islam S, Mohammed I, Naina Mohamed I, Hanna PA, Hajj SE, Jamaluddin NAH, Salameh P, Roques C (2021). Surveillance of antimicrobial resistance in low- and middle-income countries: a scattered picture. Antimicrob Resist Infect Control.

[11] Khursheed N, Adnan F, Khan MA, Hatif R (2025). Regional insights on the prevalence and antimicrobial susceptibility pattern of carbapenem and colistin-resistant gram-negative bacteria: an observational cross-sectional study from Karachi, Pakistan. BMC Infect Dis.

[12] Qureshi S, Maria N, Zeeshan M, Irfan S, Qamar FN (2021). Prevalence and risk factors associated with multi-drug resistant organisms (MDRO) carriage among pediatric patients at the time of admission in a tertiary care hospital of a developing country. A cross-sectional study. BMC Infect Dis.

[13] Murray JL, Leung DT, Hanson OR, Ahmed SM, Pavia AT, Khan AI, Szymczak JE, Vaughn VM, Patel PK, Biswas D, Watt MH (2024). Drivers of inappropriate use of antimicrobials in South Asia: A systematic review of qualitative literature. PLOS Glob Public Health.

[14] Balkan II, Alkan M, Aygün G, Kuşkucu M, Ankaralı H, Karagöz A, Şen S, Arsu HY, Biçer M, Kaya SY, Karaali R, Mete B, Saltoğlu N, Tabak F (2021). Colistin resistance increases 28-day mortality in bloodstream infections due to carbapenem-resistant Klebsiella pneumoniae. Eur J Clin Microbiol Infect Dis.

